# Supplementary material for: B cell class switch recombination is regulated by DYRK1A through MSH6 phosphorylation
Source: Nat Commun. 2023 Mar 16;14:1462. doi: 10.1038/s41467-023-37205-5 (PMC10020581; doi:10.1038/s41467-023-37205-5)
Supplement: Supplementary file 1 — Supplementary Information [file 41467_2023_37205_MOESM1_ESM.pdf]

B cell class switch recombination is regulated by DYRK1A through MSH6 phosphorylation

Liat Stoler-Barak[1], Ethan Harris[2], Ayelet Peres[3], Hadas Hezroni[1], Mirela Kuka[4], Pietro Di Lucia[4], Amalie Grenov[1], Neta Gurwicz[1], Meital Kupervaser[5], Bon Ham Yip[2], Matteo Iannacone[4,6], Gur Yaari[3], John D. Crispino[2] and Ziv Shulman[1]

[1] Department of Systems Immunology, Weizmann Institute of Science, Rehovot 761001, Israel.
[2] Department of Hematology, St. Jude Children's Research Hospital, Memphis, TN, 38105, USA.
[3] Faculty of Engineering, Bar Ilan University, Ramat Gan 52900, Israel.
[4] Vita-Salute San Raffaele University and Division of Immunology, Transplantation and Infectious Diseases, IRCCS San Raffaele Scientific Institute, Milan, Italy
[5] De Botton Institute for Proteomics, Grand Israel National Center for Personalized Medicine, Weizmann Institute of Science, Rehovot, Israel.
[6] Experimental Imaging Center, IRCCS San Raffaele Scientific Institute, Milan, Italy

Supplementary Fig. 1

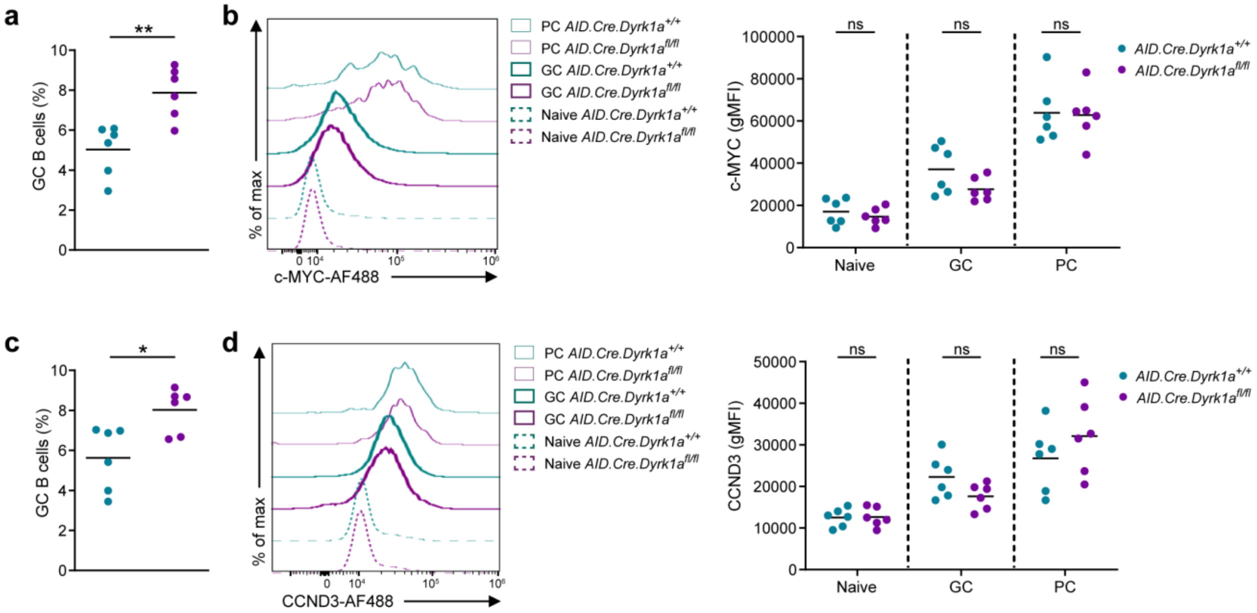

Supplementary Fig. 1. c-Myc and Cyclin D3 expression in B cells is not dependent on DYRK1A. (a, c) Frequencies of GC B cells in popliteal LNs 7 days after NP-KLH immunization (n=6; two independent experiments, two-tailed Student's t-test). **(b, d)** Representative flow cytometry histograms and frequencies of c-Myc or Cyclin D3 expression in naive, GC and plasma B cells in popliteal LNs 7 days after NP-KLH immunization (n=6; two independent experiments, two-way ANOVA). Each dot in the graphs represents a single mouse; * P=0.05, ** P≤0.01, ns, not significant.

Supplementary Fig. 2

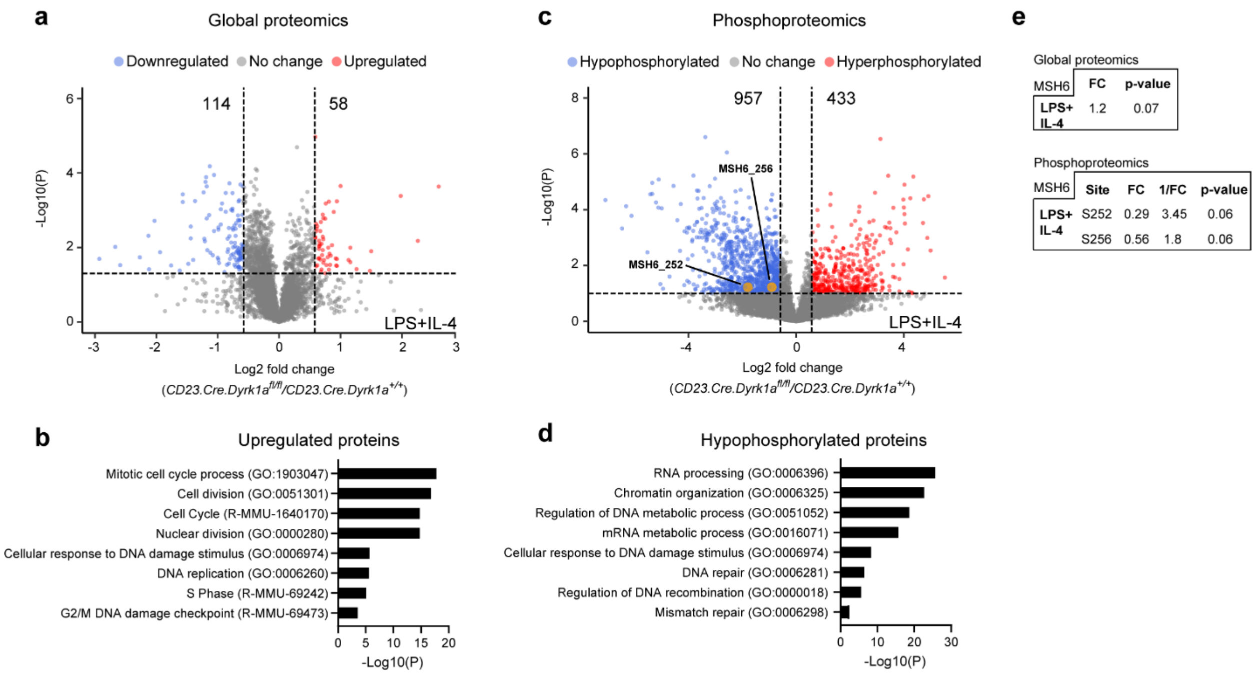

Supplementary Fig. 2. Proteomic analysis following B cell stimulation with LPS+IL-4. (a, b) Volcano plot depicting changes in protein expression in *Dyrk1a*-deficient B cell mice compared to littermates that were stimulated with LPS+IL-4 for 3 days (a), and biological pathway analysis performed on upregulated proteins (b); (Hypergeometric test and Benjamini-Hochberg p-value correction by Metascape). **(c, d)** Volcano plot showing changes in specific phosphorylation sites (c) and biological pathway analysis performed on hypophosphorylated sites in B cells stimulated with LPS+IL-4 for 3 days (d); (n=3; two independent experiments, Hypergeometric test and Benjamini-Hochberg p-value correction by Metascape). Colored points correspond to p value < 0.1 and log2 FC > 0.58 (red) or <-0.58 (blue). **(e)** Table listing detected MSH6 global (top), and phosphoproteomic (bottom) changes (two-tailed Student's t-test).

Supplementary Fig. 3

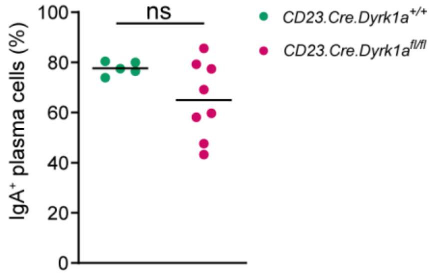

Supplementary Fig. 3. Frequency of IgA⁺ PCs in the BM. Frequencies of IgA⁺ BM PCs in unmanipulated mice (n=5-8; three independent experiments, two-tailed Student's t-test) determined by flow cytometry. Each dot in the graph represents a single mouse; ns, not significant.

Supplementary Fig. 4

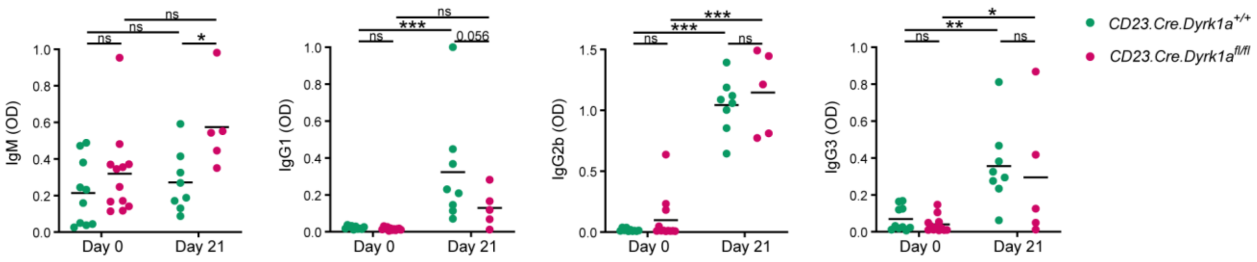

Supplementary Fig. 4. **Antibody titers of surviving mice following VSV infection.** Serum IgM, IgG1, IgG2b and IgG3 titers as determined by ELISA (n=5-8; two independent experiments, one-way ANOVA). Each dot in the graphs represents a single mouse; * P=0.05, ** P≤0.01, ***P≤0.001; ns, not significant.

Supplementary Fig. 5

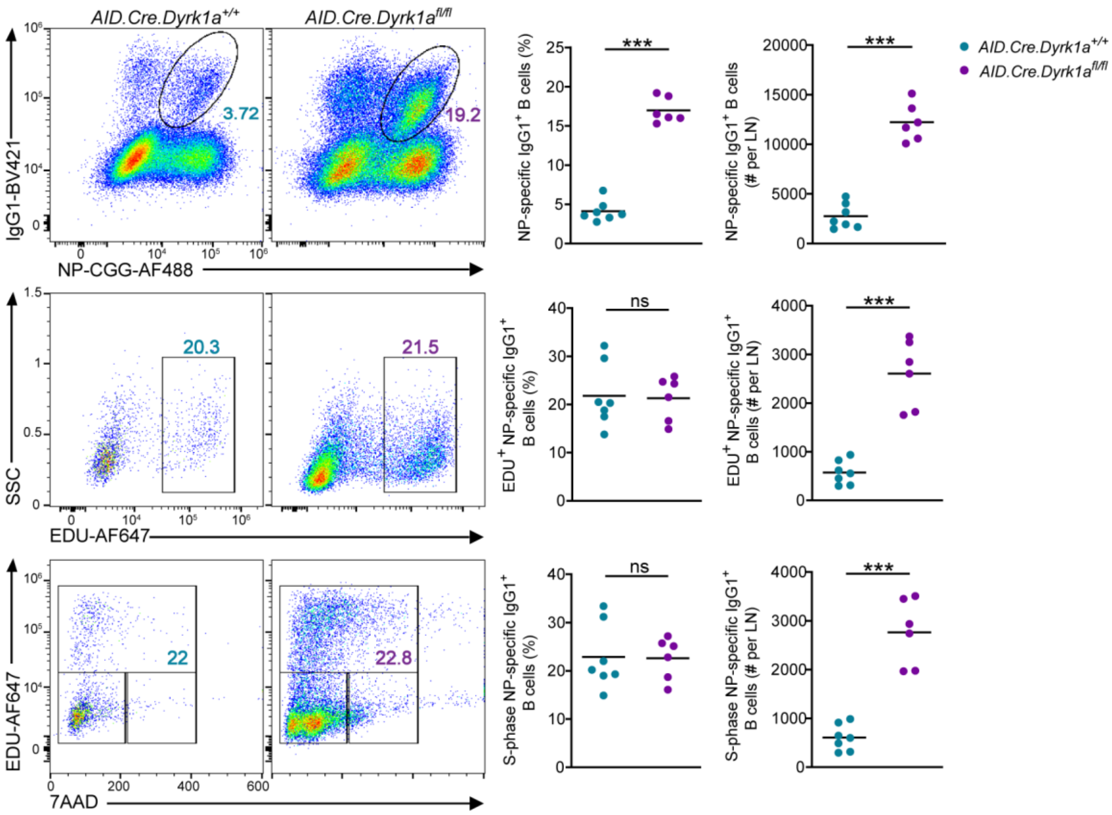

Supplementary Fig. 5. **DYRK1A restricts GC seeding by antigen-specific B cells.** Representative flow cytometry plots and frequencies or absolute numbers per single LN of NP-specific IgG1^{+} GC B cells in popliteal LNs, 7 days after NP-KLH immunization (n=6-7; two independent experiments, two-tailed Student's t-test). Each dot in the graphs represents a single mouse;***P≤0.001, ns, not significant.

Supplementary Fig. 6

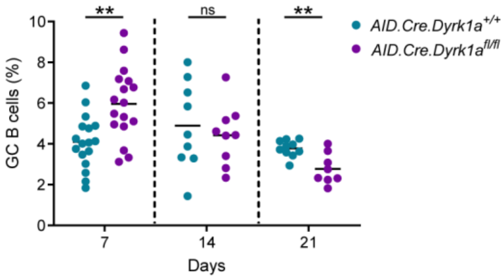

Supplementary Fig. 6. **DYRK1A restricts GC size at the early stages after immunization.** Frequencies of GC B cells in popliteal LNs 7, 14 and 21 days after NP-KLH immunization (n=8-17; four independent experiments, two-way ANOVA). Each dot in the graphs represents a single mouse;** P≤0.01, ns, not significant.

LPS+IL-4 3 days			
Substitution		*CD23.Cre.Dyrk1a*$^{+/+}$ 186 sequences 22 mutations	*CD23.Cre.Dyrk1a*$^{fl/fl}$ 175 sequences 9 mutations
		#	#
A to:	G	1	0
	T	0	0
	C	0	0
T to:	C	0	0
	A	3	0
	G	0	0
G to:	A	3	1
	T	4	0
	C	0	1
C to:	T	3	2
	A	7	3
	G	1	2

Supplementary Fig. 7. Effect of *Dyrk1a*-deficiency on the mutation landscape of the upstream Sμ-region. Analysis of the number of base substitutions in B cells stimulated with LPS+IL-4 for 3 days; n=2.

Supplementary Fig. 8

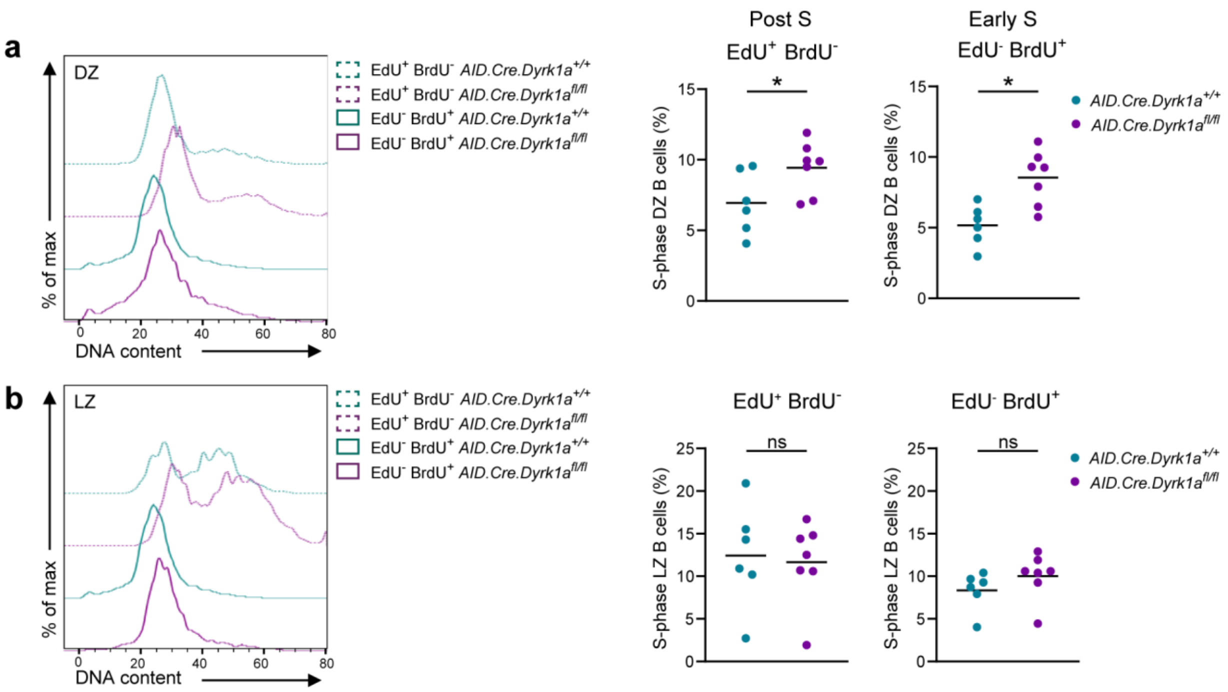

Supplementary Fig. 8. DYRK1A regulates B cell proliferation in the DZ. (a, b) Analysis of the different cell cycle stages in DZ and LZ B cells by EdU, followed by BrdU incorporation and 7AAD DNA staining 7 days after NP-KLH immunization (n=6-7; two independent experiments, two-tailed Student's t-test). Each dot in the graph represents a single mouse; * P=0.05, ns, not significant.

Supplementary Fig. 9

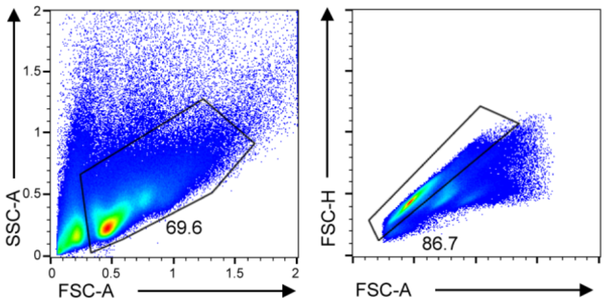

Supplementary Fig. 9. Gating strategy. Exemplary sample showing the gating strategy for identifying the lymphocytes (FSC-A vs. SSC-A), which are further gated for single cells (FSC-A vs. FSC-H).
