# Supplementary material for: B cell class switch recombination is regulated by DYRK1A through MSH6 phosphorylation
Source: Nat Commun. 2023 Mar 16;14:1462. doi: 10.1038/s41467-023-37205-5 (PMC10020581; doi:10.1038/s41467-023-37205-5)
Supplement: Supplementary file 3 — Reporting Summary [file 41467_2023_37205_MOESM3_ESM.pdf]

nature portfolio

Corresponding author(s): Ziv Shulman

Last updated by author(s): Feb 21, 2023

Reporting Summary

Nature Portfolio wishes to improve the reproducibility of the work that we publish. This form provides structure for consistency and transparency in reporting. For further information on Nature Portfolio policies, see our Editorial Policies and the Editorial Policy Checklist.

Statistics

For all statistical analyses, confirm that the following items are present in the figure legend, table legend, main text, or Methods section.

n/a	Confirmed	
☐	☒	The exact sample size (n) for each experimental group/condition, given as a discrete number and unit of measurement
☐	☒	A statement on whether measurements were taken from distinct samples or whether the same sample was measured repeatedly
☐	☒	The statistical test(s) used AND whether they are one- or two-sided<br>*Only common tests should be described solely by name; describe more complex techniques in the Methods section.*
☐	☒	A description of all covariates tested
☐	☒	A description of any assumptions or corrections, such as tests of normality and adjustment for multiple comparisons
☐	☒	A full description of the statistical parameters including central tendency (e.g. means) or other basic estimates (e.g. regression coefficient) AND variation (e.g. standard deviation) or associated estimates of uncertainty (e.g. confidence intervals)
☐	☒	For null hypothesis testing, the test statistic (e.g. F, t, r) with confidence intervals, effect sizes, degrees of freedom and P value noted<br>*Give P values as exact values whenever suitable.*
☐	☒	For Bayesian analysis, information on the choice of priors and Markov chain Monte Carlo settings
☐	☒	For hierarchical and complex designs, identification of the appropriate level for tests and full reporting of outcomes
☐	☒	Estimates of effect sizes (e.g. Cohen's d, Pearson's r), indicating how they were calculated

Our web collection on statistics for biologists contains articles on many of the points above.

Software and code

Policy information about availability of computer code

Data collection	Cytoflex software 2.5
Data analysis	Analysis was done using Perseus v1.6.2.3, Change-O v 1.2.0, NCBI IgBlast v1.17.0, Alakazam v1.2.0, SHazaM v1.1.0, R v4.2.0, MaxQuant v1.6.6.0, GSEA 4.1, UTAP pipeline v1.10.2 and Metascape v3.5.20230101

For manuscripts utilizing custom algorithms or software that are central to the research but not yet described in published literature, software must be made available to editors and reviewers. We strongly encourage code deposition in a community repository (e.g. GitHub). See the Nature Portfolio guidelines for submitting code & software for further information.

Data

Policy information about availability of data

All manuscripts must include a data availability statement. This statement should provide the following information, where applicable:

- Accession codes, unique identifiers, or web links for publicly available datasets
- A description of any restrictions on data availability
- For clinical datasets or third party data, please ensure that the statement adheres to our policy

The mass spectrometry proteomics data generated in this study have been deposited in the ProteomeXchange Consortium via the PRIDE partner repository, with the dataset identifier PXD034156 (http://proteomecentral.proteomexchange.org/cgi/GetDataset?ID=PXD034156). Proteomic data processing was used using the Uniprot human proteome database (https://www.uniprot.org/proteomes/UP000005640). The single-cell RNA sequencing data generated in this study were deposited in the NCBI's Gene Expression Omnibus database, with the dataset identifier GSE206146 (https://www.ncbi.nlm.nih.gov/geo/query/acc.cgi?

acc=GSE206146). IgH sequences were aligned to the IMGT mouse heavy chain gene database using NCBI IgBlast (https://www.ncbi.nlm.nih.gov/igblast/). Gene names were aligned to the Molecular Signature Database hallmark gene sets (http://www.gsea-msigdb.org/gsea/msigdb/human/collections.jsp#H). The remaining data are available within the paper, Supplementary Information, or Source Data file. Source data are provided together with this paper.

Human research participants

Policy information about studies involving human research participants and Sex and Gender in Research.

Reporting on sex and gender	*Use the terms sex (biological attribute) and gender (shaped by social and cultural circumstances) carefully in order to avoid confusing both terms. Indicate if findings apply to only one sex or gender; describe whether sex and gender were considered in study design whether sex and/or gender was determined based on self-reporting or assigned and methods used. Provide in the source data disaggregated sex and gender data where this information has been collected, and consent has been obtained for sharing of individual-level data; provide overall numbers in this Reporting Summary. Please state if this information has not been collected. Report sex- and gender-based analyses where performed, justify reasons for lack of sex- and gender-based analysis.*
Population characteristics	*Describe the covariate-relevant population characteristics of the human research participants (e.g. age, genotypic information, past and current diagnosis and treatment categories). If you filled out the behavioural & social sciences study design questions and have nothing to add here, write "See above."*
Recruitment	*Describe how participants were recruited. Outline any potential self-selection bias or other biases that may be present and how these are likely to impact results.*
Ethics oversight	*Identify the organization(s) that approved the study protocol.*

Note that full information on the approval of the study protocol must also be provided in the manuscript.

Field-specific reporting

Please select the one below that is the best fit for your research. If you are not sure, read the appropriate sections before making your selection.

☒ Life sciences ☐ Behavioural & social sciences ☐ Ecological, evolutionary & environmental sciences

For a reference copy of the document with all sections, see nature.com/documents/nr-reporting-summary-flat.pdf

Life sciences study design

All studies must disclose on these points even when the disclosure is negative.

Sample size	Sample sizes are sufficient to draw a conclusion. Typically and based on previous studies, at least 5 mice in two to three experiments are sufficient to test our hypotheses. Using significantly more mice is not required to reach statistical significance and unethical.
Data exclusions	On rare occasions data points were excluded. These include FACS experiments with improper EDU or BrdU labeling which indicate a technical failure in administration of these nucleotides into the mice. In the proteomic assay, out of 10 total repeats per group, 2 samples did not show similar changes in proteins and pathways as the 8 samples and were excluded.
Replication	Each experiment was conducted at least twice on different days with multiple biological repeats to ensure the reproducibility of the findings. All replication attempts were successful.
Randomization	Mice were allocated into experimental groups based on their genotyping profile, either WT or knockout.
Blinding	The investigators were not blinded to the WT or knockout mice group allocation as the investigators performed the classification into the different groups.

Reporting for specific materials, systems and methods

We require information from authors about some types of materials, experimental systems and methods used in many studies. Here, indicate whether each material, system or method listed is relevant to your study. If you are not sure if a list item applies to your research, read the appropriate section before selecting a response.

Materials & experimental systems

n/a	Involved in the study
	x Antibodies
	x Eukaryotic cell lines
x	Palaeontology and archaeology
	x Animals and other organisms
x	Clinical data
x	Dual use research of concern

Methods

n/a	Involved in the study
	ChIP-seq
	x Flow cytometry
	MRI-based neuroimaging

Antibodies

Antibodies used

Flow cytometry
CD45R/B220-APC-e780 1:400 RA3-6B2 Invitrogen 47-0452-82
CD38-AF700 1:400 90 Invitrogen 56-0381-82
CD95/FAS-PE/Cy7 1:400 Jo2 BD Biosciences 557653
IgM-APC-e710 1:400 II/41 eBioscience 46-5790-82
IgG1-FITC 1:400 RMG1-1 Biolegend 406606
IgG1-BV421 1:400 RMG1-1 Biolegend 406616
IgG2a/b-APC 1:400 X-57 Miltenyi Biotec 130-117-523
IgG3-Biotin 1:400 RMG3-1 Biolegend 406803
IgA-FITC 1:400 RMA-1 Biolegend 11-4204-81
CD138-BV605 1:400 281-2 Biolegend 142516
CD86-APC 1:400 GL-1 Biolegend 105012
CXCR4-BV421 1:400 L276F12 Biolegend 146511
c-Myc 1:400 D84C12 Cell Signaling CTS-5605S
CCND3 1:400 SP207 Abcam ab245734
Streptavidin-APC-e780 1:400 Invitrogen 47-4317-82
Goat anti-rabbit AF488 1:400 Abcam ab150077
Western blot
Beta-Actin 1:1000 D6A8 Cell Signaling CTS-84575
DYRK1A 1:1000 D30C10 Cell Signaling CTS-87655
DYRK1A 1:1000 7D10 Abnova H00001859-M01
MSH6 1:1000 OTI5D1 Origene TA807929
Thiophosphate ester 1:5000 51-8 Abcam ab92570
HRP anti mouse IgG 1:10000 NXA931 Cytiva NXA931
HRP anti rabbit 1:10000 NA934 Cytiva NA934
ELISA
goat anti-mouse IgM (ab97230), IgA (ab97235), IgG1 (ab97240) and IgG2b (ab97250)–horseradish peroxidase (Abcam) at 1:2500

Validation

Antibody validation was based on the manufacturer's statement in the data sheet, and on previous studies performed in our lab showing similar results. The manufacturer demonstrate antibody binding to immune cells using FACS plots and Western blots.

Eukaryotic cell lines

Policy information about cell lines and Sex and Gender in Research

Cell line source(s)	Cell Biolabs
Authentication	Non of the cell used were authenticated.
Mycoplasma contamination	The cell line was not tested for mycoplasma
Commonly misidentified lines (See ICLAC register)	None

Animals and other research organisms

Policy information about studies involving animals; ARRIVE guidelines recommended for reporting animal research, and Sex and Gender in Research

Laboratory animals	C57B/6 Dyrk1aflox/flox, AicdaCre/+ , Rosa26flox-stop-flox-tdTomato ,CD23cre mice at the age of 6–12 weeks. (NP KLH is a vaccination which was given to these mice).
Wild animals	The study did not involve wild animals captured in the field.

Reporting on sex	Sex was nor considered in the animal design as comparable effects were observed in our lab for both sexes.
Field-collected samples	The study did not involve samples collected in the field
Ethics oversight	All experiments on mice were approved by the Weizmann Institute Animal Care and Use Committee.

Note that full information on the approval of the study protocol must also be provided in the manuscript.

ChIP-seq

Data deposition

☐ Confirm that both raw and final processed data have been deposited in a public database such as GEO.

☐ Confirm that you have deposited or provided access to graph files (e.g. BED files) for the called peaks.

Data access links<br>*May remain private before publication.*	*For "Initial submission" or "Revised version" documents, provide reviewer access links. For your "Final submission" document, provide a link to the deposited data.*
Files in database submission	*Provide a list of all files available in the database submission.*
Genome browser session<br>(e.g. UCSC)	*Provide a link to an anonymized genome browser session for "Initial submission" and "Revised version" documents only, to enable peer review. Write "no longer applicable" for "Final submission" documents.*

Methodology

Replicates	*Describe the experimental replicates, specifying number, type and replicate agreement.*
Sequencing depth	*Describe the sequencing depth for each experiment, providing the total number of reads, uniquely mapped reads, length of reads and whether they were paired- or single-end.*
Antibodies	*Describe the antibodies used for the ChIP-seq experiments; as applicable, provide supplier name, catalog number, clone name, and lot number.*
Peak calling parameters	*Specify the command line program and parameters used for read mapping and peak calling, including the ChIP, control and index files used.*
Data quality	*Describe the methods used to ensure data quality in full detail, including how many peaks are at FDR 5% and above 5-fold enrichment.*
Software	*Describe the software used to collect and analyze the ChIP-seq data. For custom code that has been deposited into a community repository, provide accession details.*

Flow Cytometry

Plots

Confirm that:

☒ The axis labels state the marker and fluorochrome used (e.g. CD4-FITC).

☒ The axis scales are clearly visible. Include numbers along axes only for bottom left plot of group (a 'group' is an analysis of identical markers).

☒ All plots are contour plots with outliers or pseudocolor plots.

☒ A numerical value for number of cells or percentage (with statistics) is provided.

Methodology

Sample preparation	Popliteal LNs were removed, washed in cold PBS, and forced through a 70 µm mesh into PBS containing 2% FCS and 1 mM EDTA to create single-cell suspensions. Cells were subsequently incubated with fluorescently labeled antibodies for 30 min on ice. Intracellular antibody staining was performed after fixation and permeabilization with Fixation/Permeabilization Solution Kit (BD Biosciences).
Instrument	CytoFlex flow cytometer (Beckman Coulter)<br>FACS ARIA cell sorter (BD)
Software	CytoFlex<br>DIVA<br>FlowJo
Cell population abundance	We shown as percentage of total B cells or GC B cells
Gating strategy	FSC/SSC was determined based on the lymphocyte gate, followed by a singlet gate using FSC-A/FSC-H. Positive and negative

gates were based on the fluorophore intensity separating the two populations apart.

☒ Tick this box to confirm that a figure exemplifying the gating strategy is provided in the Supplementary Information.

Magnetic resonance imaging

Experimental design

Design type	*Indicate task or resting state; event-related or block design.*
Design specifications	*Specify the number of blocks, trials or experimental units per session and/or subject, and specify the length of each trial or block (if trials are blocked) and interval between trials.*
Behavioral performance measures	*State number and/or type of variables recorded (e.g. correct button press, response time) and what statistics were used to establish that the subjects were performing the task as expected (e.g. mean, range, and/or standard deviation across subjects).*

Acquisition

Imaging type(s)	*Specify: functional, structural, diffusion, perfusion.*
Field strength	*Specify in Tesla*
Sequence & imaging parameters	*Specify the pulse sequence type (gradient echo, spin echo, etc.), imaging type (EPI, spiral, etc.), field of view, matrix size, slice thickness, orientation and TE/TR/flip angle.*
Area of acquisition	*State whether a whole brain scan was used OR define the area of acquisition, describing how the region was determined.*

Diffusion MRI ☐ Used ☐ Not used

Preprocessing

Preprocessing software	*Provide detail on software version and revision number and on specific parameters (model/functions, brain extraction, segmentation, smoothing kernel size, etc.).*
Normalization	*If data were normalized/standardized, describe the approach(es): specify linear or non-linear and define image types used for transformation OR indicate that data were not normalized and explain rationale for lack of normalization.*
Normalization template	*Describe the template used for normalization/transformation, specifying subject space or group standardized space (e.g. original Talairach, MNI305, ICBM152) OR indicate that the data were not normalized.*
Noise and artifact removal	*Describe your procedure(s) for artifact and structured noise removal, specifying motion parameters, tissue signals and physiological signals (heart rate, respiration).*
Volume censoring	*Define your software and/or method and criteria for volume censoring, and state the extent of such censoring.*

Statistical modeling & inference

Model type and settings	*Specify type (mass univariate, multivariate, RSA, predictive, etc.) and describe essential details of the model at the first and second levels (e.g. fixed, random or mixed effects; drift or auto-correlation).*
Effect(s) tested	*Define precise effect in terms of the task or stimulus conditions instead of psychological concepts and indicate whether ANOVA or factorial designs were used.*

Specify type of analysis: ☐ Whole brain ☐ ROI-based ☐ Both

Statistic type for inference (See Eklund et al. 2016)	*Specify voxel-wise or cluster-wise and report all relevant parameters for cluster-wise methods.*
Correction	*Describe the type of correction and how it is obtained for multiple comparisons (e.g. FWE, FDR, permutation or Monte Carlo).*

Models & analysis

n/a	Involved in the study
☐	☐ Functional and/or effective connectivity
☐	☐ Graph analysis
☐	☐ Multivariate modeling or predictive analysis

Functional and/or effective connectivity	*Report the measures of dependence used and the model details (e.g. Pearson correlation, partial correlation, mutual information).*
Graph analysis	*Report the dependent variable and connectivity measure, specifying weighted graph or binarized graph, subject- or group-level, and the global and/or node summaries used (e.g. clustering coefficient, efficiency, etc.).*
Multivariate modeling and predictive analysis	*Specify independent variables, features extraction and dimension reduction, model, training and evaluation metrics.*
